# Brown adipose tissue and regulation of human body weight

**DOI:** 10.1002/dmrr.3594

**Published:** 2022-11-22

**Authors:** Elissa Harb, Omar Kheder, Gishani Poopalasingam, Razi Rashid, Akash Srinivasan, Chioma Izzi‐Engbeaya

**Affiliations:** ^1^ Imperial College School of Medicine Imperial College London London UK; ^2^ Section of Endocrinology and Investigative Medicine Imperial College London London UK

**Keywords:** brown adipose tissue (BAT), capsaicin, capsinoids, cold activation, energy expenditure, farnesoid X receptor, mirabegron, obesity, thermogenesis

## Abstract

**Background:**

Approximately 30% of the global population is affected by obesity. Traditional non‐surgical measures for weight loss have limited efficacy and tolerability. Therefore, there is a need for novel, effective therapies. Brown adipose tissue (BAT) has been implicated in physiological energy expenditure, indicating that it could be targeted to achieve weight loss in humans. The use of ^18^F‐fluorodeoxyglucose (^18^F‐FDG) positron emission tomography—computed tomography—(PET‐CT) imaging has enabled the discovery of functionally active BAT in the supraclavicular, subclavian, and thoracic spine regions of human adults. This review aims to discuss the reasons behind the renewed interest in BAT, assess whether it is metabolically important in humans, and evaluate its feasibility as a therapeutic target for treating obesity.

**Sources of material:**

PubMed Central, Europe PMC, Medline.

**Findings:**

*In vivo* studies have shown that BAT activity is regulated by thyroid hormones and the sympathetic nervous system. Furthermore, BAT uniquely contains uncoupling protein 1 (UCP1) that is largely responsible for non‐shivering thermogenesis. Cold exposure can increase BAT recruitment through the browning of white adipose tissue (WAT); however, this technique has practical limitations that may preclude its use. Currently available medicines for humans, such as the β3‐adrenergic receptor agonist mirabegron or the farnesoid X receptor agonist obeticholic acid, have generated excitement, although adverse effects are a concern. Capsinoids represent a tolerable alternative, which require further investigation.

**Conclusions:**

The use of currently available BAT‐activating agents alone is unlikely to achieve significant weight loss in humans. A combination of BAT activation with physical exercise and modern, successful dietary strategies represents a more realistic option.

## 1 BACKGROUND

The global prevalence of obesity has been increasing exponentially over the last few decades. In 2014, over 2.1 billion individuals were estimated to be overweight or obese, accounting for approximately 30% of the world's population.^1^ Due to the numerous diseases associated with increased adiposity including type 2 diabetes and cancer, the economic burden of obesity is substantial^1^ and thus there is an urgent need for novel effective treatments.

Bariatric surgery is currently the most effective intervention for achieving weight loss, but it is accompanied by the costs of specialist equipment, inpatient stay, and the requirement for appropriately trained and specialised healthcare providers.^2^ Moreover, not every obese individual is eligible for and/or chooses surgery due to peri‐operative and longer‐term complications, and there is limited availability of bariatric surgeons.^3^ Therefore, there is a requirement for effective and accessible medical therapies.

Obesity is the consequence of a long‐term imbalance between energy intake and energy expenditure; therefore, drugs have been developed to address this imbalance. For example, liraglutide is a glucagon‐like peptide‐1 (GLP1) agonist, which enhances satiety and delays gastric emptying, thus reducing calorie intake.^4^ This therapeutic agent is effective but nausea, constipation, and pancreatitis are potential side‐effects.^5^ Conversely, monoamine reuptake inhibitors, such as sibutramine, cause weight loss by increasing energy expenditure, but the substantial risk of adverse cardiovascular outcomes resulted in its withdrawal from the market.^6^ Hence, there is still a need for additional safe and tolerable pharmacological agents to treat obesity.

Researchers have recently shifted their focus towards brown adipose tissue (BAT), which represents an alternative, potentially safer therapeutic target for increasing energy expenditure. Whilst white adipose tissue (WAT) is responsible for storing energy in the form of triglycerides, BAT has been implicated in physiological energy expenditure through a process called non‐shivering thermogenesis.^7^ It has therefore been hypothesised that increasing BAT recruitment and activation may result in upregulated energy expenditure and consequent weight loss. Furthermore, studies suggest that WAT can be converted into thermogenic beige adipocytes in a process known as browning, which may represent an alternative therapeutic approach.^8^ This review will discuss the reasons behind the renewed interest in BAT, assess whether it is metabolically important in humans, and evaluate the feasibility of BAT activation and WAT browning in the treatment of obesity.

## 2 BROWN ADIPOSE TISSUE (BAT)

**FIGURE 1:**
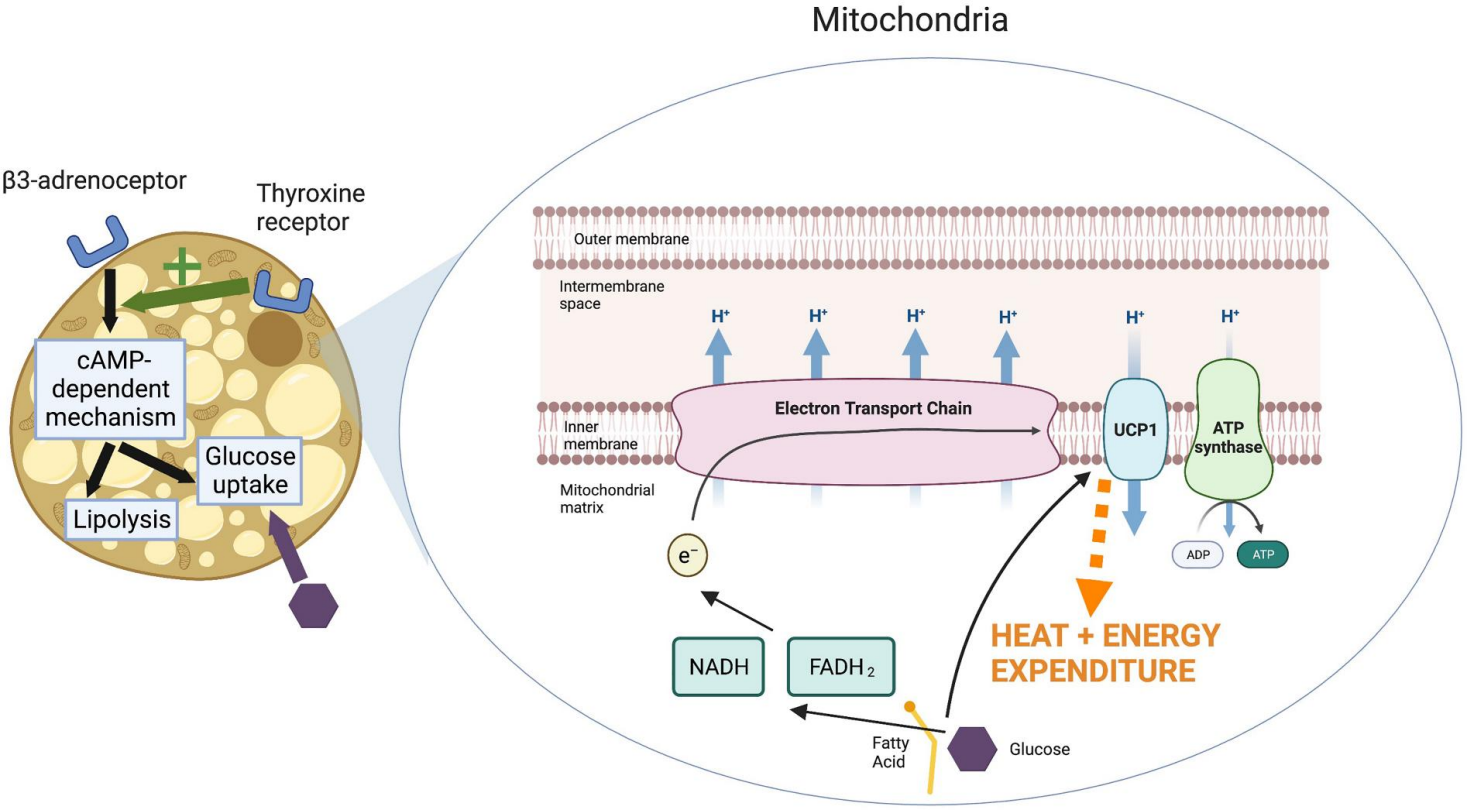
Thermogenesis in the mitochondria of brown adipose tissue. Increased sympathetic activity and β3‐adrenoceptor stimulation drives a cAMP‐dependent mechanism, resulting in glucose uptake and lipolysis of intracellular triglycerides. Thyroxine receptor stimulation enhances the effects of increased sympathetic activity. Fatty acids and glucose are utilised in the electron transport chain and activate UCP1, which uncouples the respiratory chain from the ATP synthesis by providing an alternative route for the passage of H^+^. The flow of H^+^ through UCP1 generates heat via non‐shivering thermogenesis. Created with BioRender.com. ADP, adenosine diphosphate; ATP, adenosine triphosphate; cAMP, cyclic adenosine monophosphate; e^−^, electron; FADH_2_, reduced flavin adenine dinucleotide; H^+^, proton; NADH, reduced nicotinamide adenine dinucleotide; UCP1, uncoupling protein 1

BAT is distinct from WAT in both structure and function^7^, ^9^, ^10^; its constituent adipocytes are characterised by numerous mitochondria that give BAT its characteristic brown colour.^11^ These mitochondria contain uncoupling protein 1 (UCP1), which is responsible for the role of BAT in thermogenesis and increasing energy expenditure.^11^ When BAT is activated, fatty acids are utilised in the electron transport chain and UCP1 acts as an alternative channel to adenosine triphosphate (ATP) synthase for the passage of protons, thereby releasing the energy as heat instead of producing ATP (Figure 1).^12^

Consequently, the concept of therapeutically stimulating BAT, in order to increase energy expenditure by utilising fat, is of great interest to the scientific community. Glucose is also taken up by BAT, and therefore, enhanced BAT activation is likely to improve glucose metabolism and benefit patients with diabetes, which is a common comorbidity in obesity.^13^

**FIGURE 2:**
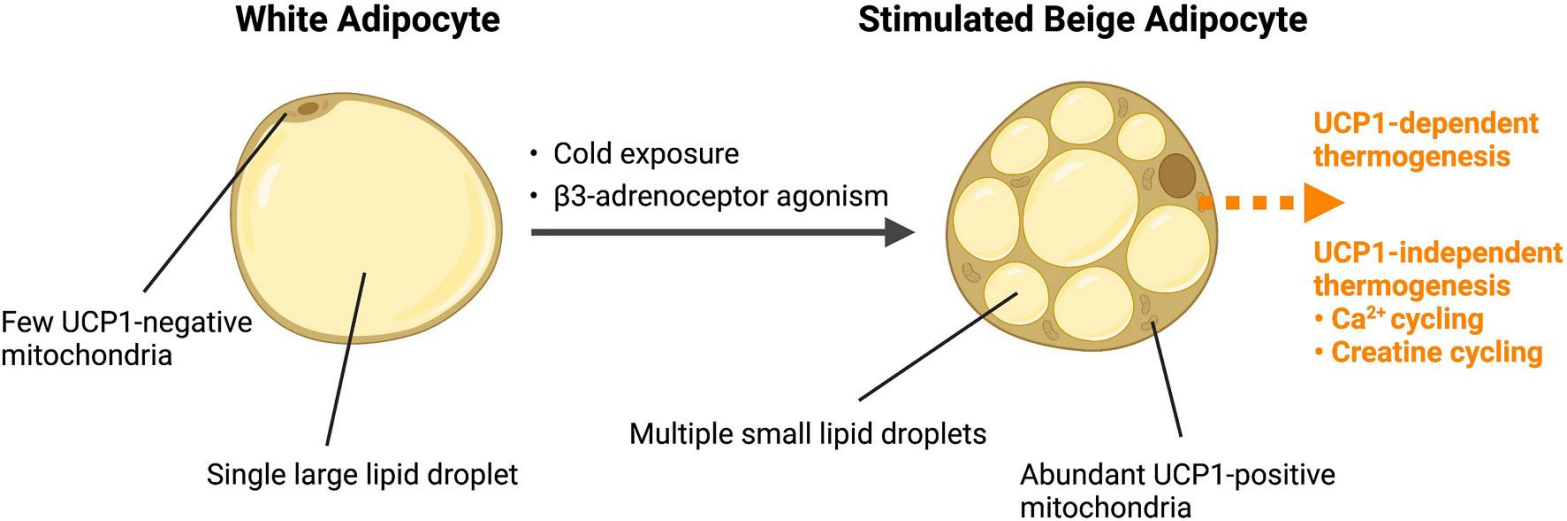
Beiging of white adipose tissue. Cold exposure and β3‐adrenoceptor agonism converts white adipocytes into stimulated beige adipocytes, which contain UCP1‐positive mitochondria. Beige adipocytes can undergo thermogenesis via UCP1‐dependent and UCP1‐independent mechanisms. Created with BioRender.com. Ca^2+^, calcium ion; UCP1, uncoupling protein 1

Alongside white and brown adipocytes, scientists have discovered other adipocytes known as beige adipocytes (Figure 2). These cells typically exist within WAT depots, although data indicates that they may also be present alongside brown cells in BAT tissue.^14^ Beige adipocytes express UCP1 in their mitochondria and thus can undertake thermogenesis.^15^ Despite the similarities in the structures of brown and beige adipocytes, there are notable differences in their function. Brown adipocytes express high levels of UCP1 under normal conditions, whereas beige cells only express UCP1 in response to external stimuli, such as cold exposure or β3‐adrenoceptor agonism.^12^ Beige cells may also undergo thermogenesis via UCP1‐independent mechanisms, such as ATP‐dependent calcium cycling^16^ or creatine cycling.^17^ Additionally, the two types of cell have contrasting developmental lineages: brown adipocytes are derived from a specific subset of myogenic factor 5 positive dermatomyotome cells, whereas beige adipocytes classically arise in WAT depots and are derived from platelet‐derived growth factor α positive precursors.^18^, ^19^ The key similarities and differences between brown and beige adipocytes are summarised in Table 1. It has been reported that WAT can be converted into beige adipose tissue,^8^, ^20^ which could represent an additional approach for treating obesity.

**TABLE 1:** Summary of the key similarities and differences between brown and beige adipocytes

	Brown adipocytes	Beige adipocytes
Location	Present in supraclavicular, subclavian, and thoracic spine regions.	Arise in white adipose tissue depots in response to external stimuli.
Cellular structure	Several small fat droplets. Abundant mitochondria and UCP1.	Single fat droplet, with multiple smaller droplets after stimulation. Abundant mitochondria and UCP1 after stimulation.
Origin	Myogenic factor 5 positive precursors.	Platelet‐derived growth factor α positive precursors.
Thermogenesis	UCP1‐dependent.	UCP1‐dependent. UCP1‐independent (Ca^2+^ cycling and creatine cycling).

Abbreviations: Ca^2+^, calcium ion; UCP1, uncoupling protein 1.

## 3 BROWN FAT IN HUMAN ADULTS

Despite the implication of BAT in energy expenditure, the quantity of this tissue decreases from infancy to adulthood, and it was believed that adult humans only possessed insignificant quantities of BAT.^21^ Therefore, there was limited enthusiasm about the therapeutic activation of BAT for many years. However, recent innovations in imaging and laboratory techniques have led to fascinating discoveries about the presence and functionality of BAT in adulthood.

The gold standard imaging modality for assessing BAT activity is currently ^18^F‐fluorodeoxyglucose (^18^F‐FDG) positron emission tomography—computed tomography (PET‐CT), which measures the uptake of ^18^F‐FDG by metabolically active tissues.^22^ Through the use of this imaging technique, regions of functionally active BAT were discovered in the supraclavicular, subclavian, and thoracic spine regions of adult humans.^23^, ^24^ Early imaging studies also observed greater levels of ^18^F‐FDG uptake in female patients.^24^ Although the use of ^18^F‐FDG PET‐CT imaging identified the presence of functional BAT in adults, the quantity of BAT present in children remains higher than in adults.^24^ Subsequent studies have since corroborated these findings regarding the effects of sex^25^ and age^26^ on the presence of BAT with the prevalence of detectable BAT reported to be over 2‐fold greater in women than men^25^ and higher in younger age groups.^26^

More recently, studies have discovered additional factors that appear to affect the quantity of BAT observed, including body mass index (BMI) and seasons.^27^ ^18^F‐FDG uptake following cold stimulation was higher in the winter compared to the summer, which may be due to the colder room temperature associated with winter.^27^, ^28^ There also appears to be an inverse correlation between BAT activity and BMI, as shown by van Marken Lichtenbelt *et al.* ^29^ who found that mean BAT activity was significantly, 4‐fold higher in lean participants (BMI <25) compared to overweight or obese participants (BMI ≥25), despite non‐significant differences in BAT volume. This is supported by the finding that basal and cold‐induced fatty acid uptake by BAT is impaired in obesity.^30^ Leitner *et al.* ^31^ also reported that lean men (mean BMI = 23.2) had over twice the amount of activated BAT compared to obese men (mean BMI = 34.8). Finally, Matsushita *et al.* ^28^ found that participants with detectable BAT had lower BMI, body fat mass, and abdominal fat area compared to those without detectable BAT. However, it should be noted that blood glucose, which may have been higher in the obese participants,^29^ can compete with ^18^F‐FDG and result in lower uptake measurements.^32^

Although several ^18^F‐FDG PET‐CT imaging studies have been conducted to detect and quantify BAT in humans, it is difficult to perform objective comparisons. This is because these studies used different protocols, and experimental factors including the ambient temperature, the injected dose of ^18^F‐FDG and the time from ^18^F‐FDG injection to imaging can significantly influence the results.^33^ The use of varying standard uptake value (SUV) thresholds also limits the comparability of these studies.^23^, ^24^ In 2016, Chen *et al.* ^33^ proposed the standardised BARCIST 1.0 criteria, which should guide future imaging studies in order to improve comparability.

There are several limitations to the use of ^18^F‐FDG PET‐CT imaging. Firstly, this imaging technique does not conclusively detect the presence or absence of BAT; instead, it enables the active tissue to be visualised in a functional manner.^34^ Therefore, new methods are required to identify inactive BAT in humans. Secondly, the high ionising radiation levels mean that there are ethical concerns with using this technique in children^34^; and alternatives such as magnetic resonance imaging (MRI) could overcome this issue. Finally, the concept of ^18^F‐FDG PET‐CT imaging is based on the ability of BAT to metabolise glucose,^22^ but fatty acids are the main substrates for BAT.^12^ Lipid tracers are also capable of detecting BAT^35^ and this technology may help us to further understand the physiological roles of BAT.

Furthermore, the technique of ^18^F‐FDG PET‐CT imaging can yield false positives due to uptake of the tracer by muscles.^24^ Therefore, studies that only used imaging could not conclusively prove the existence of BAT in adult humans. However, Virtanen *et al.* ^13^ obtained biopsies from the tissues exhibiting high ^18^F‐FDG uptake before quantifying UCP1 levels, and the results were highly suggestive of BAT. These transformational findings were supported by Zingaretti *et al.*,^26^ who collected adipose tissue from the necks of surgical patients and discovered islands of UCP1‐positive brown adipocytes. Taken together,^13^, ^23^, ^26^ it can be concluded that substantial amounts of BAT are present in human adults, and that they occupy well‐defined regions.

## 4 BAT PHYSIOLOGY AND WEIGHT CONTROL

The discovery of active BAT in adults generated significant excitement in the scientific community.^13^ Nevertheless, there was a need for greater understanding about BAT physiology in order to fully uncover its therapeutic potential. Firstly, assessing the contribution of BAT thermogenesis towards overall energy expenditure is required to ascertain whether BAT activation could actually achieve meaningful weight loss. Secondly, researchers have also investigated the precise mechanisms underlying BAT activation and induction to uncover specific targets for pharmaceutical interventions. These two topics are discussed below.

### 4.1 Is BAT important for energy expenditure in humans?

Energy expenditure is predominantly driven by the basal metabolic rate, which is primarily dictated by body size and is responsible for up to 70% of total energy output. The remaining 30% is composed of the energy required for physical movement and thermogenesis.^36^

To date, a variety of strategies have been used to elicit weight loss via increased energy expenditure with limited success. For example, physical exercise is a safe and natural way of increasing energy expenditure, but numerous studies have shown that the use of exercise alone results in minimal weight loss.^37^ Thyroid hormone supplementation has also been trialled as a method of increasing the basal metabolic rate.^38^ However, this approach is associated with the severe side effects of atrial fibrillation and osteoporosis, as well as hyperphagia that limits the weight loss.^38^ Finally, recent studies have investigated an array of dietary strategies, including a meta‐analysis by Ludwig *et al.* ^39^ showed that low carbohydrate diets initially reduce total energy expenditure before increasing it in the long term. Nevertheless, diets are notoriously difficult to sustain, and they represent an unrealistic solution for many obese individuals.^40^

The contribution of BAT‐specific thermogenesis to whole‐body energy expenditure in humans has been postulated to be 5%^11^ (70–90 kcal/day) or even less than 20 kcal/day in another study.^41^ This implies that merely activating the existing BAT may not increase energy expenditure sufficiently to achieve clinically significant weight loss. However, converting WAT into BAT (or beige adipose tissue) to increase its contribution to whole‐body energy expenditure could yield more impressive results.^8^ Interestingly, Chen *et al.* ^42^ demonstrated that BAT volume in rodents was significantly increased following a Roux‐en‐Y gastric bypass, which might explain some of the weight loss benefits of bariatric surgery.

A combination of BAT activation with physical exercise and the modern, successful dietary strategies demonstrated in the DiRECT trial^43^ may be far more successful than utilising each approach separately. Moreover, several studies have suggested that exercise itself may influence BAT activation and WAT beiging. Various exercise benefits are mediated by peroxisome proliferator‐activated receptor γ co‐activator 1α (PGC1‐α),^44^ and rodent studies have linked PGC1‐α expression with the release of the hormone irisin^8^. In vitro and in vivo experiments showed that irisin induces UCP1 expression in white adipocytes, highlighting a link between exercise and WAT beiging.^8^ Notably, irisin was also present in human plasma at higher levels following exercise.^8^ In addition to WAT beiging, it has been postulated that exercise may stimulate BAT via the increased sympathetic nervous system activity that accompanies exercise, although further research is required.^45^ Taken together, the use of physical exercise alongside other BAT activation approaches may have a synergistic effect on energy expenditure and weight loss.

### 4.2 The mechanism of BAT thermogenesis

As described above, BAT contains UCP1 that can be activated by fatty acids or glucose (Figure 1).^13^ UCP1 allows protons to bypass ATP synthase and uncouples oxidative phosphorylation from ATP synthesis, thus generating heat instead of ATP in a process called thermogenesis.^12^ This process fluctuates in response to different stimuli: cold‐induced thermogenesis maintains a normal body temperature,^29^ whereas diet‐induced thermogenesis compensates for overfeeding.^11^

Furthermore, rodent BAT expresses β3‐adrenergic receptors (β3) and thyroid hormone receptors with studies demonstrating that the sympathetic nervous system acts synergistically with thyroid hormones to modulate the BAT activity.^46^ β3‐adrenoceptor stimulation by noradrenaline drives a cAMP‐dependent mechanism that breaks down intracellular triglycerides to release free fatty acids, which act as a substrate for UCP1.^47^ Another effect of sympathetic stimulation is the increased expression of the enzyme deiodinase type II, which upregulates the availability of local thyroid hormones.^46^ Conversely, the binding of thyroxine to thyroid hormone receptors enhances the effects of β3‐adrenoceptor stimulation on the BAT activity.^46^ Notably, thyroid hormones have also been shown to induce WAT browning in mice, independently of the sympathetic nervous system, although the resultant beige adipose tissue did not demonstrate increased thermogenesis due to insufficient adrenergic stimulation.^48^

Alternative mechanisms for the regulation of BAT activation have been proposed. Studies by Schreiber *et al.* ^49^ and Shin *et al.* ^50^ unexpectedly showed that the intracellular production of free fatty acids by brown adipocytes driven by noradrenaline may not be essential for UCP1 activation. Therefore, one alternative model suggests that UCP1 is activated by external fatty acids derived from WAT instead; however, this model does not explain the functional significance of the sympathetic innervation of BAT.^51^ Another proposal is that there is an unknown, non‐fatty acid, direct activator of UCP1 that is released following β3‐adrenoceptor stimulation.^51^ Further research is required to evaluate the plausibility of these alternative mechanisms.

Research has provided further insight into the molecular biology of BAT. For example, Puigserver *et al.* ^52^ showed that nuclear receptors like peroxisome‐proliferator‐activated receptor γ (PPARγ) work with the PPARγ coactivator 1α (PGC1α) to induce BAT thermogenesis. It may be possible to target these downstream targets in the future to enhance BAT activation. Meanwhile, Seale *et al.* ^53^ identified PRDM16 as a key transcriptional regulator, which promotes the formation of brown adipocytes. Therefore, PRDM16 could serve as a promising therapeutic target for increasing the abundance of BAT.

## 5 BAT AS A THERAPEUTIC TARGET FOR WEIGHT LOSS

Several studies have shown that cold exposure can increase BAT recruitment and energy expenditure in humans,^13^, ^27^, ^29^, ^54^ but it is unclear whether this would lead to significant weight loss. Three of these experiments were conducted by exposing participants to mildly cold temperatures of 16°C^29^ and 19°C^27^ for 2 h. Virtanen *et al.* ^13^ exposed patients to a room temperature of 17–19°C for 2 h, and additionally, placed the subjects' feet in ice water during the scans. Similarly, participants in the study by Yoneshiro *et al.* ^54^ experienced 2‐h cold exposure at 19°C whilst intermittently placing their legs on ice blocks. All 4 studies showed that cold exposure resulted in increased ^18^F‐FDG uptake,^13^, ^27^, ^29^, ^54^ suggesting an increase in BAT recruitment, although this effect was diminished in older participants.^27^ Moreover, a positive correlation was identified between BAT activity as per ^18^F‐FDG uptake and the measured rise in energy expenditure following cold exposure, implying that BAT is a key contributor.^54^

To achieve clinically significant weight loss, cold‐induced BAT activation would be required for longer than the 2‐h periods described above. A 2016 study demonstrated that BAT can be re‐activated and recruited in obese people by a 10‐day period of cold acclimation, which involved exposure to 14–15°C temperatures for 6‐h periods daily between days 3 and 10.^55^ Notably, there were consequent metabolic benefits observed including enhanced glucose uptake.^55^ Longer studies have also been conducted: Lee *et al.* ^56^ demonstrated that a 1‐month period of overnight temperature acclimation at 19°C significantly enhanced diet‐induced thermogenesis and postprandial insulin sensitivity. 6 weeks of 2‐h cold exposure at 17°C was trialled by Yoneshiro *et al.*,^57^ which was associated with increased BAT activity, and notably, an accompanying 5.2% reduction in body fat mass.

Despite the promising results to date, there are numerous limitations to these studies. 1‐month^56^ and 6‐week^57^ periods of cold exposure are still insufficient to achieve meaningful weight loss. Hence, future clinical trials should pilot the use of cold exposure for longer durations, or intense short bursts, and assess for a significant effect on weight loss. However, there may be issues with retaining participants in such trials; in the aforementioned studies of 1 month^56^ and 6 weeks^57^ duration, there was no dropout but only 5 and 12 subjects, respectively, underwent cold exposure, whereas participant withdrawal may become a greater issue in larger studies. Furthermore, cold exposure has been associated with an increase in free fatty acids,^58^ which could be detrimental to the cardiovascular health of obese patients.^59^

The use of cold exposure to recruit and activate BAT has provided valuable physiological insights but concerns about long‐term tolerability and adverse effects may limit its use as a therapeutic approach for weight loss. Conversely, a variety of pharmacological approaches to target BAT have been trialled in rodents and humans, with varying levels of success, and these are discussed below.

### 5.1 β3‐adrenoceptor agonism

In an in vitro study, Cao *et al.* showed that β3‐agonism stimulates WAT browning by increasing UCP1 expression.^60^ The authors demonstrated that the effects of β3 stimulation on WAT browning are mediated by the p38‐MAPK signalling pathway, as these effects were not observed when a p38‐MAPK inhibitor was administered.^60^ It is also important to note that β3‐agonism resulted in pleiotropic effects, including an increase in lipolysis and improved insulin sensitivity.^60^

Mirabegron is a β3‐agonist, which is currently licenced for treatment of bladder overactivity.^61^ Due to its presumed selectivity for β3‐adrenoceptors, researchers have studied the use of mirabegron for BAT activation in rodents^62^ and humans.^61^ In obese mice, in vivo administration of mirabegron resulted in a 12% lower body weight, reduced adiposity and 14‐fold increased gene expression of UCP1 compared to vehicle.^62^ In vitro experiments showed that mirabegron stimulates higher UCP1 expression in and browning of 3T3‐L1 white preadipocytes; increased UCP1 expression was also observed in mouse brown preadipocytes.^62^ Conversely, a very modest upregulation in UCP1 was observed in BAT in vivo,^62^ suggesting that UCP1 is almost maximally expressed in fully differentiated BAT and/or mirabegron does not increase BAT UCP1 expression. Calorimetry was not conducted in this study^62^ so it was not possible to quantify energy expenditure and assess its contribution to weight loss.

Clinical trials have provided conflicting results regarding the use of mirabegron. In 2015, Cypess *et al.* ^63^ found that administering mirabegron to healthy men for 12 weeks was associated with the higher BAT activity and the resting metabolic rate compared to participants receiving placebo. The authors calculated that the observed energy expenditure could translate to the 5 kg/year weight loss required for FDA authorisation.^63^ However, the selected 200 mg/day dose is higher than the FDA‐approved 50 mg/day dose (for bladder overactivity), and cardiovascular side‐effects such as raised blood pressure and heart rate were observed due to off‐target β1‐ and/or β2‐agonism.^63^

A more recent study corroborated these results. O’Mara *et al.* ^64^ also employed the 200 mg/day dose and found that 4‐week therapy of mirabegron led to increased BAT activity and resting energy expenditure in healthy women. Again, mild cardiovascular symptoms were reported such as headaches and palpitations.^64^ Taken together,^63^, ^64^ high‐dose mirabegron is capable of stimulating BAT in men and women, but the side‐effects indicate that an alternative with fewer off‐target effects would be more suitable due to the potential for adverse cardiovascular events with longer periods of high‐dose mirabegron administration. Neither study^63^, ^64^ demonstrated weight loss, but the promising effects on energy expenditure suggest the need for longer trials lasting at least 6 months, similar to other weight loss drug trials.^65^

Whilst high doses of mirabegron can activate BAT in healthy humans,^64^ other groups have demonstrated that the FDA‐approved 50 mg/day dose did not result in a significant increase in resting energy expenditure or weight loss in obese participants after 12 weeks.^61^ These results may be due to the inverse correlation between BAT and BMI.^29^ However, mirabegron induced beiging of subcutaneous WAT and subsequently improved β‐cell function in the insulin‐resistant participants.^61^ Therefore, this low dose of mirabegron should be administered for longer periods (e.g. 6 months) to assess whether adipose tissue beiging would eventually translate into meaningful weight loss.

An explanation for the modest performance of mirabegron in clinical trials to date was recently proposed by Blondin *et al.* ^66^ Here, they showed that the β2‐adrenoceptor mediates BAT thermogenesis in humans,^66^ unlike in rodents where β3 signalling is key.^62^ These findings suggest that β3‐agonism is unlikely to induce significant weight loss in humans, and that the high doses of mirabegron administered in the above studies^63^, ^64^ increased BAT energy expenditure via non‐selective β2‐agonism.^66^ This explanation has since been challenged in a study by Cero *et al.*,^67^ where experiments involving primary cultures of human brown/beige adipocytes demonstrated that mirabegron activates BAT specifically via β3‐agonism. Targeting β2‐adrenoceptors could still be a more effective approach of activating BAT in humans, but pre‐clinical testing would be difficult due to differential β2‐adrenoceptor distribution between rodents and humans. Furthermore, β2‐adrenoceptors are present in multiple sites in the body,^68^ and hence, chronic β2‐agonism in humans carries the risk of numerous side‐effects, including tremors and tachycardia.

### 5.2 Alternative approaches

Aside from β3‐adrenoceptor agonism, other approaches have been trialled to therapeutically target BAT. Capsaicin is the active component of chilli peppers, and it is believed to mediate the effects of chilli peppers on thermogenesis^69^ by activating BAT and stimulating the browning of WAT.^70^ However, capsaicin is also a chemical irritant, which is responsible for the pungency of chilli peppers,^69^ and hence, it is unlikely to serve as a tolerable therapeutic. Instead, Yoneshiro *et al.* ^71^ trialled the use of non‐pungent capsaicin analogues called capsinoids in humans. Capsinoids lack the prominent taste of chilli peppers, so it was possible to perform a placebo‐controlled trial. A small but significant elevation in energy expenditure was observed 1 h after the oral ingestion of capsinoids, but only in participants with detectable BAT.^71^ Therefore, capsinoids, which can be extracted from sweet chilli peppers,^72^ may serve as a tolerable alternative to capsaicin for targeting BAT.

Although capsinoids may be capable of activating BAT, the findings of Yoneshiro *et al.* ^71^ indicate that they may be ineffective as a therapeutic when BAT is reduced or absent. Consequently, they may be of limited use in obesity where the presence of BAT is thought to be diminished.^29^ However, a randomised controlled trial reported that capsinoid ingestion by participants with a mean BMI of 30.4 for 12 weeks resulted in significant abdominal fat loss.^73^ Gastrointestinal adverse events, including dyspepsia and bowel disturbances, were noted, but they were not considered serious and did not result in withdrawal.^73^ A series of in vitro and in vivo experiments then showed that capsaicin activates the transient receptor potential vanilloid 1 (TRPV1) channel, which results in the browning of WAT.^70^, ^74^ Thus, the prolonged use of capsinoids in obesity may convert WAT into beige adipose tissue, promote beige activity and increase energy expenditure to reduce abdominal adiposity.

There are additional novel therapeutics that have shown potential in rodents. Fang *et al.* ^75^ notably demonstrated that fexaramine, an intestine‐specific farnesoid X receptor (FXR) agonist, exerts numerous beneficial effects on metabolism including WAT browning. Furthermore, fexaramine administration prevented diet‐induced weight gain in obese mice.^75^ This FXR agonist was minimally absorbed from the gut^75^ (and thus may result in a lower risk of systemic toxicity), although further animal and human studies are warranted.

The metabolic effects of FXR‐agonism in mice appeared to be secondary to the induction of fibroblast growth factor 15 (Fgf15) production, which has a human orthologue called FGF19, and the consequent changes to the composition of circulating bile acids.^75^ In particular, chenodeoxycholic acid derivatives were present in higher proportions^75^ and chenodeoxycholic acid has since been shown to activate human BAT.^76^ Therefore, the findings of Fang *et al.* ^75^ may be translatable to humans and intestine‐specific FXR agonism may be an effective therapeutic approach to achieve adipose tissue browning and subsequent weight loss. Currently, obeticholic acid is an example of an FXR agonist that is currently used in humans to treat primary biliary cholangitis,^77^ and this agent has also been shown to stimulate BAT.^78^ However, its effect on bile acids can commonly result in pruritus, as demonstrated by the PBC OCA International Study of Efficacy where over 50% of participants experienced pruritus as a side‐effect.^79^ This tolerability issue may limit the use of obeticholic acid as a BAT‐stimulating agent.

Ultimately, a variety of therapeutic agents have been studied to date, but there is a lack of strong evidence to suggest that a single drug can achieve meaningful weight loss in humans via BAT activation. However, combining pharmacological agents with behavioural interventions such as exercise and diets may result in greater calorie deficits and achieve better outcomes. BAT activation may also help to address some of the limitations of behavioural interventions; for example, significant weight loss from dieting is often accompanied by a slower resting metabolic rate, thus limiting the long‐term effectiveness of the diet.^80^ Furthermore, the constrained total energy expenditure model describes the adaptation of the human body to increased physical activity by limiting energy expenditure on other physiological processes.^81^ The use of pharmacological agents to activate BAT could counteract these metabolic adaptations to lifestyle modifications and help sustain the benefits of physical activity and diets, although long‐term clinical trials are necessary. Adverse effects are another key issue for many therapeutic agents, but strategies combining the use of multiple drugs may enable lower dosing to reduce the likelihood of severe toxicities. Nevertheless, this approach carries the risk of drug interactions, and thus further research is required.

## 6 CONCLUSION

It is evident that adult humans possess significant quantities of BAT^13^; yet the feasibility of utilising activated BAT for effecting significant weight loss in humans remains unclear. The portion of total energy expenditure that can be attributed to BAT activity in humans appears to be low,^11^, ^41^ and hence, BAT activation alone is unlikely to be a successful weight loss strategy. Additionally, BAT has been found in lean, healthy individuals but it is less detectable in the obese individuals that are the target patient group.^29^ Further investigation is warranted to elucidate whether a lack of BAT causes an increase in weight or vice versa, in order to gain valuable insight into the pathophysiology of obesity.

As we learn more about the physiology of human BAT, therapeutics will continue to be developed and trialled. The β3‐agonism approach using a currently available medicine, mirabegron, has shown the greatest potential to date.^63^ However, this was associated with adverse cardiovascular side‐effects due to off‐target effects.^64^ Furthermore, a greater understanding about the differences between rodent and human BAT will be necessary to improve upon the modest clinical trial results that have been observed so far.^66^ A promising alternative strategy involves FXR agonism using obeticholic acid, although this is also limited by side‐effects.^79^ Ingesting non‐pungent capsinoids to activate BAT may be the most tolerable approach,^71^ but further studies are required to assess whether clinically significant weight loss can be achieved in this manner.

The abundance of BAT in humans is inversely correlated with age^26^ and BMI,^29^ hence merely activating the existing BAT is unlikely to achieve significant weight loss in the patients who need it the most. Drugs that are capable of converting WAT into beige adipose tissue may prove to be more successful in achieving meaningful weight loss, although there is still a lack of conclusive evidence as to whether this can be done safely. There are suggestions that BAT volume may be increased after bariatric surgery,^42^ and thus, BAT activation could be used to potentiate and sustain the weight loss benefits of surgery. Nevertheless, this suggestion requires confirmation in human studies. Consequently, the most realistic option for the near future is a combination of tolerable BAT‐activating agents with physical exercise to maximise total energy expenditure, alongside healthy hypocaloric diets to achieve weight loss.

## AUTHOR CONTRIBUTIONS

Conceptualization: Akash Srinivasan, Elissa Harb, Omar Kheder, Gishani Poopalasingam, Razi Rashid, and Chioma Izzi‐Engbeaya; Writing—original draft, reviewing and editing: Akash Srinivasan, Elissa Harb, Omar Kheder, Gishani Poopalasingam, Razi Rashid, and Chioma Izzi‐Engbeaya; Final approval: Akash Srinivasan, Elissa Harb, Omar Kheder, Gishani Poopalasingam, Razi Rashid, and Chioma Izzi‐Engbeaya.

## CONFLICTS OF INTEREST

The authors declare no conflicts of interest.

### PEER REVIEW

The peer review history for this article is available at https://publons.com/publon/10.1002/dmrr.3594.

## ETHICS STATEMENT

Not applicable.

## Data Availability

Data sharing not applicable to this article as no datasets were generated or analysed during the current study.
